# Exogenous Cysteine Improves Mercury Uptake and Tolerance in *Arabidopsis* by Regulating the Expression of Heavy Metal Chelators and Antioxidative Enzymes

**DOI:** 10.3389/fpls.2022.898247

**Published:** 2022-06-10

**Authors:** Yeon-Ok Kim, Yonghyun Gwon, Jangho Kim

**Affiliations:** ^1^Interdisciplinary Program in IT-Bio Convergence System, Chonnam National University, Gwangju, South Korea; ^2^Department of Convergence Biosystems Engineering, Chonnam National University, Gwangju, South Korea; ^3^Department of Rural and Biosystems Engineering, Chonnam National University, Gwangju, South Korea

**Keywords:** cysteine, mercury uptake, transporter, heavy metal chelator, antioxidative enzyme, gene expression

## Abstract

Cysteine (Cys) is an essential amino acid component of the major heavy metal chelators, such as glutathione (GSH), metallothioneins (MTs), and phytochelatins (PCs), which are involved in the pathways of mercury (Hg) tolerance in plants. However, the mechanism through which Cys facilitates Hg tolerance in plants remains largely unclear. In this study, we investigated the effects of exogenous Cys on Hg uptake in the seedlings, roots, and shoots of *Arabidopsis* throughout 6 and 36 h of Hg exposure and on the regulation of Hg detoxification by heavy metal chelators and antioxidative enzymes. The results showed that exogenous Cys significantly improved Hg tolerance during the germination and seedling growth stages in *Arabidopsis*. Exogenous Cys significantly promoted Hg uptake in *Arabidopsis* roots by upregulating the expression of the Cys transporter gene *AtLHT1*, resulting in increased Hg accumulation in the roots and seedlings. In *Arabidopsis* seedlings, exogenous Cys further increased the Hg-induced glutathione synthase (*GS1* and *GS2*) transcript levels, and the Hg and Hg + Cys treatments greatly upregulated *MT3* expression after 36 h exposure. In the roots, *MT3* was also significantly upregulated by treatment of 36 h of Hg or Hg + Cys. Notably, in the shoots, *MT2a* expression was rapidly induced (10-fold) in Hg presence and further markedly increased (20-fold) by exogenous Cys. Moreover, in the seedlings, exogenous Cys upregulated the transcripts of all superoxide dismutase (*CuSOD1*, *CuSOD2*, *MnSOD1*, *FeSOD1*, *FeSOD2*, and *FeSOD3*) within 6 h and subsequently increased the Hg-induced *GR1* and *GR2* transcript levels at 36 h, all of which could eliminate the promotion of reactive oxygen species production and cell damage caused by Hg. Additionally, exogenous Cys upregulated all the antioxidative genes rapidly in the roots and subsequently increased the expression of *CuSOD1*, *CuSOD2*, and *MnSOD1* in the shoots. These results indicate that exogenous Cys regulates the transcript levels of heavy metal chelators and antioxidative enzymes differently in a time- and organ-specific manner under Hg stress. Taken together, our study elucidates the positive functional roles of exogenous Cys in the Hg uptake and tolerance mechanisms of *Arabidopsis*.

## Introduction

Natural or human activities, such as mining, wastewater discharge, fertilizer use, and waste incineration, have resulted in the wide dispersal of mercury (Hg) in the environment (Patra and Sharma, 2000; Wang and Greger, 2004; Beckers and Rinklebe, 2017; Natasha et al., 2020). This heavy metal pollutant has no biological role in the physiology of living organisms, including plants and animals, and is in fact toxic and deadly if absorbed into the organ system (Tangahu et al., 2011; Gworek et al., 2020). As plants are the initial primary organisms in the hierarchy of terrestrial ecosystems, the accumulation of Hg would be a major threat to human health through the food chain. In this regard, phytoremediation is a potential strategy for the removal of toxic Hg from contaminated soils by accumulating, chelating, or transforming the heavy metal into biologically inactive forms *via* Hg-tolerant plants.

Mercury in soil is easily absorbed by the plant roots, wherein the intracellular Hg ions will react directly with the sulfhydryl (–SH) groups of vital enzymes, proteins, and other biomolecules, thereby disrupting the protein structures, the enzyme activities, and the proper functioning of various physiological activities, such as water flow and mineral nutrient uptake, stomatal conductance, and photosynthesis, resulting in diminished plant growth and yields (Zhang and Tyerman, 1999; Patra and Sharma, 2000; Pisani et al., 2011; Chen et al., 2012; Ajsuvakova et al., 2020). Therefore, to achieve successful phytoremediation of soils through the production of Hg hyperaccumulators, it is important to understand the mechanisms of Hg accumulation and tolerance in plants, for example, the regulation of Hg uptake and exclusion, chelation, vacuolar compartmentalization, and reactive oxygen species (ROS) homeostasis (Natasha et al., 2020).

Once heavy metals are taken up by the roots, plants accelerate the generation of various low-molecular-weight (LMW) thiols, such as cysteine (Cys), glutathione (GSH), phytochelatins (PCs), and metallothioneins (MTs) (Anjum et al., 2015; Hasan et al., 2017), which possess high binding affinity toward toxic metals. Heavy metal chelation *via* LMW thiols is the primary mechanism for heavy metal detoxification at the cellular level and can alleviate the disruption of cellular metabolism by these toxic pollutants (Küpper and Kroneck, 2005; Hasan et al., 2017). Cys, a sulfur-containing amino acid, is surmised to play a crucial role in Hg chelation because its side chain that contatins –SH group has a particularly strong binding affinity toward Hg ions (Ajsuvakova et al., 2020). Furthermore, Cys occupies a central position in the major LMW thiols, such as GSH, PCs, and MTs. Cys is a precursor molecule for the synthesis of GSH (γ-Glu-Cys-Gly) by GSH synthase (GS), and GSH is subsequently converted to PCs [(γ-Glu-Cys)*n*-Gly (*n* = 2–11)] through phytochelatin synthase (PCS) activity (Cobbett and Goldsbrough, 2002; Yadav, 2010; Zagorchev et al., 2013). It has been demonstrated that the Hg-binding ability of GSH, PCs, and MTs is carried out by the –SH group of the Cys residues present in each metal chelator (Wu and Wang, 2012; Kim et al., 2017; Ajsuvakova et al., 2020). In addition to the *in vitro* evidence, *in vivo* studies have shown that GSH, PCs, and MTs form complexes with Hg ions in the plant cytosol, where they are sequestered into vacuoles, thereby allowing plant cells to avoid the toxic effect of the heavy metal (Hasan et al., 2017; Natasha et al., 2020; Zhao et al., 2022). However, there are yet no reports that have clearly described the effects of exogenous Cys on the uptake and internal fate of Hg in plants, such as its chelation, transportation, and detoxification.

Despite sufficient evidence demonstrating the effects of GSH, PCs, and MTs on heavy metal accumulation and tolerance (*viz.*, through their strong abilities to bind such pollutants), relatively few studies about the role of Cys in these processes have been reported. Increased Cys biosynthesis has been shown to affect heavy metal tolerance and accumulation in several plants such as *Arabidopsis*, tobacco, brassica, and poplar (Nakamura et al., 2009; Ning et al., 2010; Jia et al., 2016; Rajab et al., 2020). However, there are hardly any reports documenting whether these same effects can be achieved through the supplementation of exogenous Cys to plants. Recent studies have shown that exogenous Cys treatment could increase lead and chromium tolerance in *Iris* and maize seedlings, even under the condition of high accumulation of these two heavy metals (Yuan et al., 2015; Terzi and Yıldız, 2021). Additionally, exogenous Cys increased the tolerance of maize plants to Hg but also decreased Hg accumulation (Aysin et al., 2020). Although these studies have clearly pointed to the importance of Cys in heavy metal tolerance—despite the different patterns of heavy metal accumulation observed according to the heavy metal type and plant species—our current knowledge on the potential role that exogenous Cys plays in the plant mechanism of Hg accumulation under Hg stress remains limited.

Hg promotes the plant cell production of excess amounts of ROS, such as superoxide ($O_{2}^{-}$) and hydrogen peroxide (H_2_O_2_), which cause oxidative stress-induced cell damage that eventually results in metabolic disruptions and plant growth restriction (Ortega-Villasante et al., 2007; Shiyab et al., 2009; Meng et al., 2011; Kim et al., 2017; Natasha et al., 2020). Plants have developed antioxidative systems comprising nonenzymatic antioxidants (e.g., amino acids, GSH, ascorbic acid, phenolic compounds, flavonoids, α-tocopherols, etc.) and enzymatic antioxidants [*viz.*, superoxide dismutase (SOD), peroxidase, catalase (CAT), ascorbate peroxidase (APX), and glutathione reductase (GR)] to counteract the excess ROS (Mittler, 2002; Foyer and Noctor, 2003; Mittler et al., 2004). Many studies have demonstrated the importance of the activation of enzymatic antioxidants as a plant adaptation response to Hg-induced oxidative stress (Zhou et al., 2007; Shiyab et al., 2009; Priya et al., 2014; Malar et al., 2015). There is clear evidence that Cys itself has ROS-scavenging activity, such as that of $O_{2}^{-}$ and H_2_O_2_ (Paulsen and Carroll, 2013; Kim et al., 2020). Furthermore, it has been shown that the application of exogenous Cys decreased the activities of CAT and APX in cobalt-stressed *Ocimum basilicum* (Azarakhsh et al., 2015) but increased those of CAT and POD in chromium-treated maize (Terzi and Yıldız, 2021). In a study by Aysin et al. (2020), the treatment of Hg-stressed maize with exogenous Cys increased the expression of SOD and CAT as well as the activities of SOD, peroxidase, and CAT but not those of APX and GR. As there is scant information available to link Cys to ROS scavenging in plants during their response to Hg stress, the elucidation of the role that this amino acid plays in the regulation of antioxidative enzymes in plants under Hg stress would add valuable knowledge to this field of study.

Although previous lines of evidence have indicated the important role of Cys in heavy metal tolerance in plants, the cellular role of exogenous Cys and the molecular mechanisms underlying its control of Hg uptake and intracellular Hg detoxification in *Arabidopsis* plants under Hg stress remain unclear. In this study, we investigated the effects of exogenous Cys supplementation on Hg uptake and on the regulation of LMW thiols (GSH, PC, and MT) and antioxidative enzyme expression during 6 h (short exposure) and 36 h (long exposure) of Hg exposure in *Arabidopsis* seedlings as well as in roots and shoots of the reproductive phase. The results of this study could serve as a reference on the possible exogenous Cys-mediated mechanisms of Hg tolerance.

## Materials and Methods

### Plant Materials and Phenotype Analysis

To determine the optimum concentrations of Cys to use in this study, seeds of *Arabidopsis thaliana* (Col-0) were surface sterilized with 5% sodium hypochlorite, rinsed in sterile distilled water, and sown on Murashige–Skoog (MS) agar medium containing different concentrations of Cys (25, 50, or 100 μM) for phenotype analysis. *Arabidopsis* seeds were germinated on half-strength MS agar medium containing HgCl_2_ (10, 20, and 30 μM) with or without Cys and then grown vertically in a growth chamber at 22°C under a 16 h light/8 h dark photoperiod. The seedling length was recorded every day during the Hg stress period.

For the estimation of root elongation, *Arabidopsis* seedlings of similar size were transferred to MS medium supplemented with Hg or Hg + Cys and grown vertically, following which the primary root lengths were measured.

### Treatments and Experimental Design

*Arabidopsis* seeds were allowed to germinate and grow for 14 d on filter paper supplemented with MS agar medium and then exposed to a solution containing 20 μM Hg with or without 50 μM Cys for 6 and 36 h. The samples were used for the assessment of various physiological parameters and gene expression profiles. For analysis of the roots and shoots, *Arabidopsis* seedlings of similar size were cultured in Hoagland’s nutrient solution, after which 3-week-old plants (reproductive phase) were treated as indicated above. The roots and shoots were then separated for further analysis. All experiments were performed at least three times.

### Determination of Hg Content

Young seedlings and plants in the reproductive phase were treated with Hg or Hg + Cys for 6 and 36 h, and then thoroughly washed with H_2_O. The roots and shoots of 3-week-old *Arabidopsis* plants were also separated. All samples were dried at 80°C until completely dehydrated and their Hg content was then determined using a DMA-80 direct mercury analyzer (Milestone, Italy).

### Determination of H_2_O_2_ and Cell Damage Levels

The H_2_O_2_ content was measured according to the procedure described by Kim et al. (2017), with slight modifications. In brief, *Arabidopsis* seedlings that had been treated either with or without 50 μM Cys in the presence of 20 μM Hg were homogenized in 0.1% (w/v) trichloroacetic acid, after which the homogenate was centrifuged at 10,000 *g* for 20 min at 4°C. The supernatant was added to a reaction mixture consisting of 0.5 ml of 100 mM potassium phosphate buffer (pH 7.0) and 1 ml of 1 M potassium iodide. After incubation for 1 h in the dark, the H_2_O_2_ content in the mixture was determined by referencing a standard curve generated with known H_2_O_2_ concentrations, with monitoring of the absorbance at 410 nm.

For the detection of cell death, *Arabidopsis* seedlings were treated with 20 μM Hg with or without 50 μM Cys for 36 h and the roots of *Arabidopsis* seedlings were first stained with 0.25% (w/v) Evans blue solution for 10 min and then washed thrice with water (Zhang et al., 2015). Finally, the stained roots were photographed.

### Quantitative Real-Time PCR Expression Analysis

Total RNA extraction and purification were carried out using an RNeasy Plant Mini kit (Qiagen, Valencia, CA, United States). To quantitate the mRNA expression levels of genes encoding transporters, heavy metal chelators, and antioxidative enzymes, the quantitative reverse transcription polymerase chain reaction (qRT-PCR) was conducted using a Rotor-Gene Q PCR cycler (Qiagen) and QuantiTect SYBR Green RT-PCR kit (Qiagen) as described previously (Kim and Kang, 2018). Actin was used as the internal control. The primers used for the qRT-PCR analysis are listed in Supplementary Tables 1 and 2. At least three independent replicates were performed for all experiments with different RNA samples.

### Statistical Analysis

All experiments were performed at least three times. The effects of Hg stress and Cys treatment on all tested plant parameters were analyzed by ANOVA followed by Student’s *t*-test analysis (SIGMAPLOT Software; Systat Software Inc.). Statistically significant differences were defined as *p* < 0.05.

## Results

### Effects of Exogenous Cys on Germination and Seedling Growth Under Hg Stress

**Figure 1:**
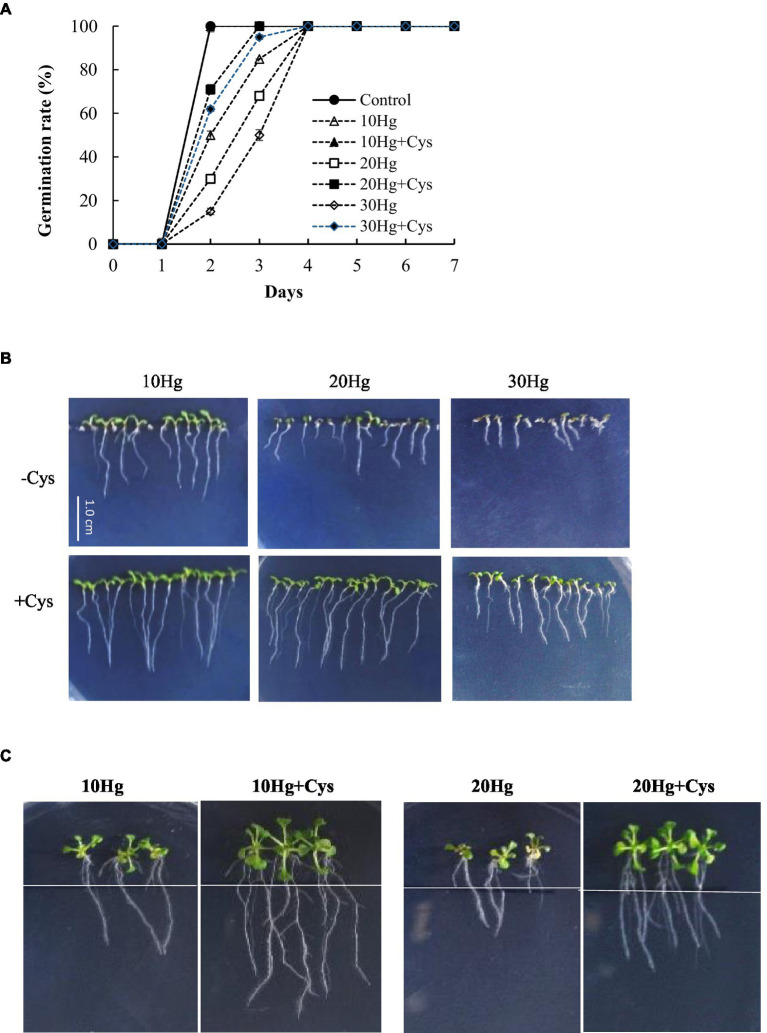
Effects of exogenous Cys on the germination rate **(A)** and post-germination seedling growth **(B)** and root growth of *Arabidopsis* **(C)** under Hg stress. Germination and post-germination seedling growth were observed on MS medium supplemented with HgCl_2_ (10–30 μM) with or without 50 μM Cys. Root growth was investigated after transfer of five-day-old seedlings to MS medium containing 10 or 20 μM Hg with or without 50 μM Cys.

We investigated whether Cys plays a positive role in Hg tolerance during germination and seedling growth in various plants. To select the appropriate concentration of Cys to apply, the effects of different concentrations (0, 25, 50, or 100 μM) of the amino acid on *Arabidopsis* seed germination and seedling growth were tested (Supplementary Figure 1). Seed germination was not affected by the different amino acid concentrations up to 50 μM but was inhibited by a concentration of 100 μM Cys. However, slight inhibition of post-germination seedling growth was found with 50 μM Cys. Therefore, 50 μM Cys was selected for the subsequent experiments to elucidate its effects on Hg tolerance in plants. Although the germination rate and post-germination seedling growth were significantly decreased with the increase in Hg concentration alone, the co-treatment with exogenous 50 μM Cys improved both these processes at all indicated treatment durations under 10, 20, or 30 μM Hg (Figures 1A,B, and Supplementary Table 3). Moreover, in the presence of Cys, the aerial tissue was also larger and greener than that under 30 μM Hg exposure alone (Figure 1B). To evaluate the effect of Cys on root growth, 5-day-old *Arabidopsis* seedlings were transferred to agar medium containing Hg or Hg + Cys (Figure 1C and Supplementary Table 3), whereupon 50 μM Cys were found to have greatly alleviated the Hg-induced inhibition of root growth.

### Effects of Exogenous Cys on Hg Accumulation in *Arabidopsis*

**Figure 2:**
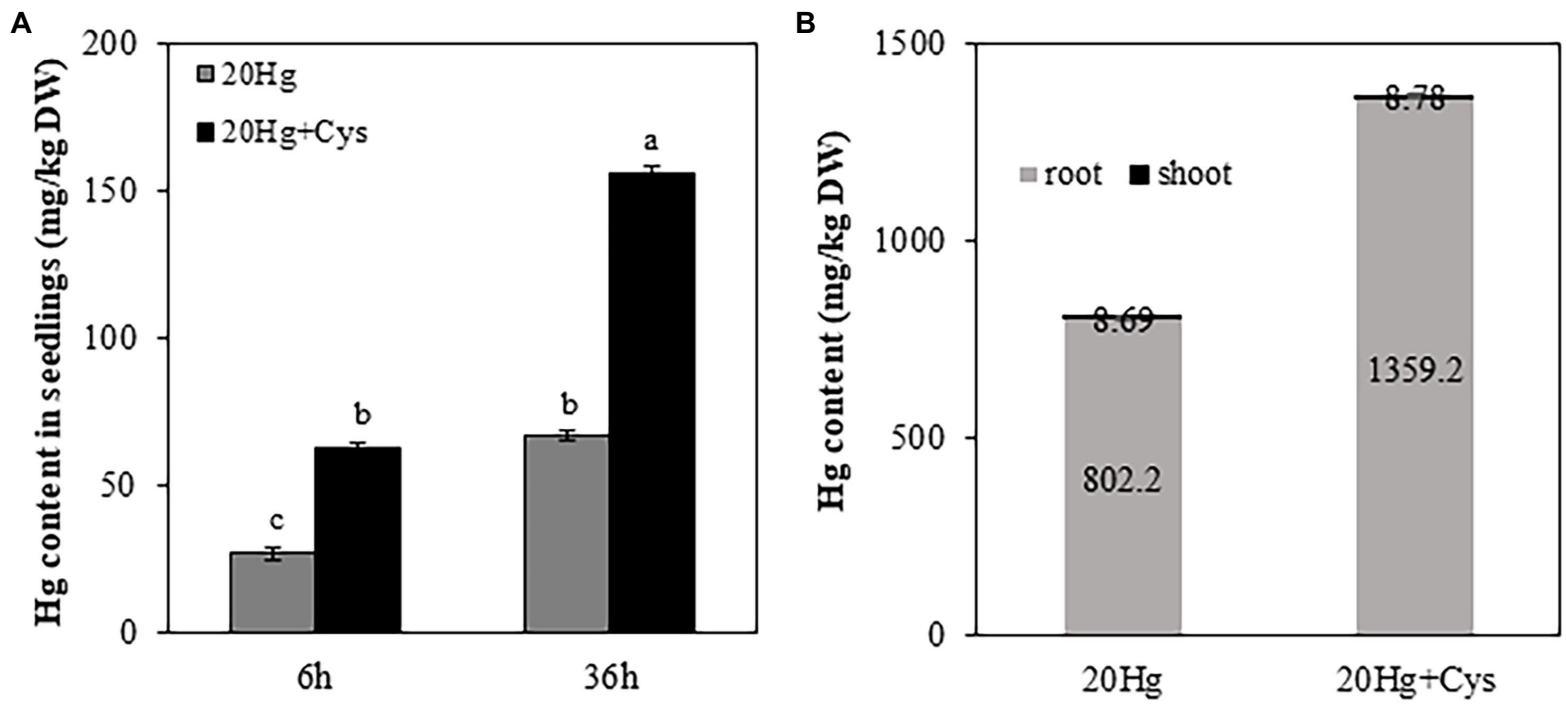
Effects of exogenous Cys on Hg accumulation in seedlings **(A)** and different organs (roots and shoots; **B**) of *Arabidopsis* under Hg stress. Ten-day-old seedlings were treated with 20 μM Hg with or without 50 μM Cys for 6 and 36 h. Hg content in roots and shoots from 3-week-old *Arabidopsis* was determined after treatment of 20 μM Hg with or without 50 μM Cys for 36 h. Data represent the mean ± SD of three independent replicates. Different letters indicate significant differences (*p* < 0.05).

To determine the effect of exogenous Cys supplementation on Hg accumulation in *Arabidopsis*, the seedlings were treated with 20 μM Hg with or without Cys for 6 and 36 h (Figure 2A). Cys treatment increased Hg accumulation in the seedlings by almost 2-fold of that under Hg exposure alone at both 6 and 36 h. Additionally, the increase in Hg accumulation was time dependent, being more than 2-fold higher at 36 h than at 6 h under both Hg and Hg + Cys treatment conditions.

To elucidate the Hg distribution pattern in *Arabidopsis*, the concentration of Hg was determined in roots and shoots from the reproductive phase. A significantly higher Hg concentration (> 100-fold) was observed in the roots than in the shoots, regardless of whether the plants had been treated with Hg or Hg + Cys (Figure 2B). Interestingly, exogenous Cys increased Hg accumulation much more in the roots but did not alter it in the shoots.

### Effects of Exogenous Cys on Hg Uptake by *Arabidopsis* Roots

**Figure 3:**
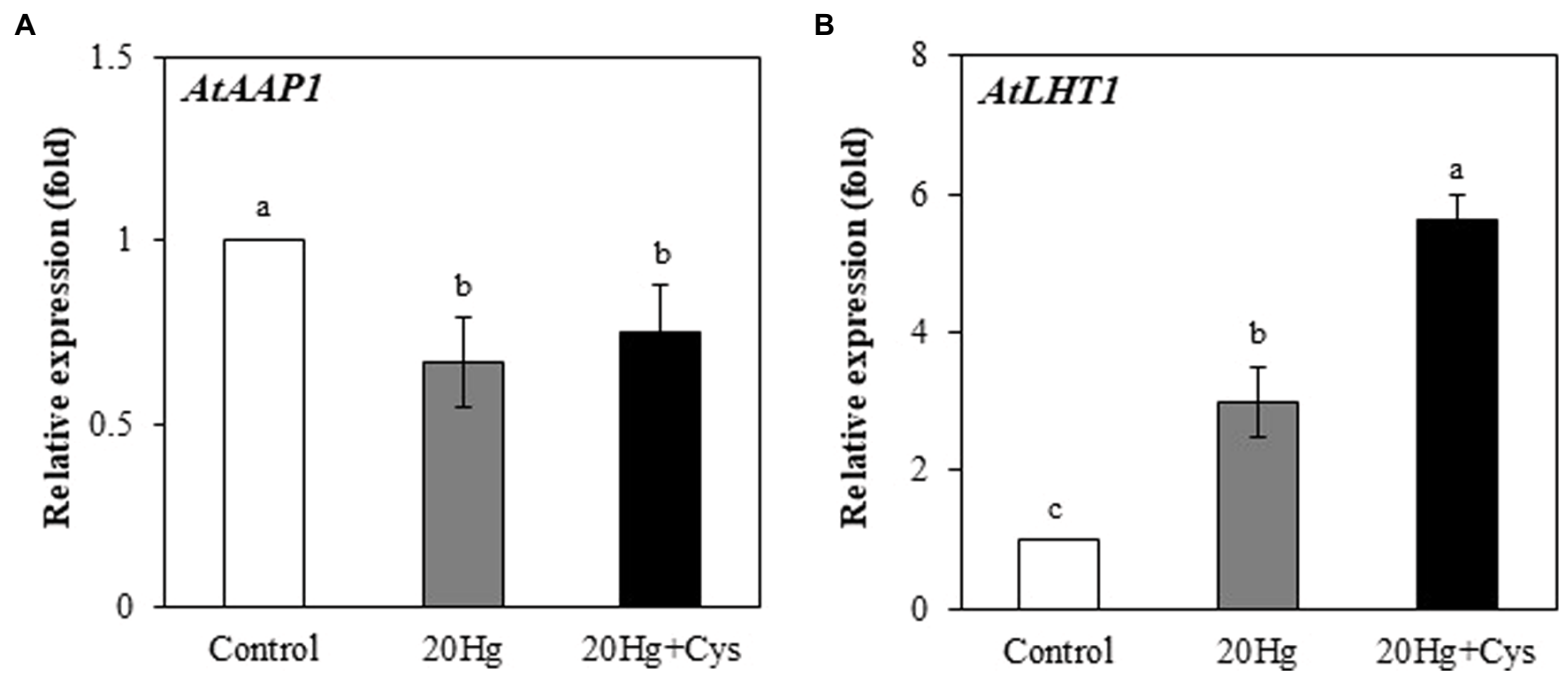
Effects of exogenous Cys on the expression levels of *AtAPP1* **(A)** and *AtLHT1* **(B)** in *Arabidopsis* roots under Hg stress. Three-week-old *Arabidopsis* was treated with 20 μM Hg with or without 50 μM Cys for 36 h, and roots were collected for one replication. Data represent the mean ± SD of three independent replicates. Different letters indicate significant differences (*p* < 0.05).

It is important to investigate how exogenous Cys enhances Hg accumulation. ^1^H NMR spectroscopy analysis showed a large shift of two protons, H1 and H2 in Cys solution containing Hg (Supplementary Figure 2). ^1^H NMR spectra revealed that Cys was tightly bound to the Hg ion at the –SH group of its side chain, indicating that Hg was transported into the root cells *via* its complexation with Cys. To ascertain the role of Cys in Hg uptake, we determined whether it regulated the transcripts of *A. thaliana* amino acid permease 1 (*AtAAP1*) and lysine histidine transporter 1 (*AtLHT1*), which are known root membrane-associated transporters for neutral amino acids such as Cys (Himer et al., 2006; Lee et al., 2007; Perchlik et al., 2014). The *AtAAP1* transcript levels in the roots were slightly decreased in the presence of Hg, regardless of whether or not exogenous Cys was present. By contrast, the *AtLHT1* transcript level was notably increased (2.99-fold) under Hg treatment alone and was even more significantly increased (5.61-fold) in the presence of exogenous Cys (Figures 3A,B).

### Effects of Exogenous Cys on Transcript Levels of Heavy Metal Chelators in Seedlings Under Hg Stress

**Figure 4:**
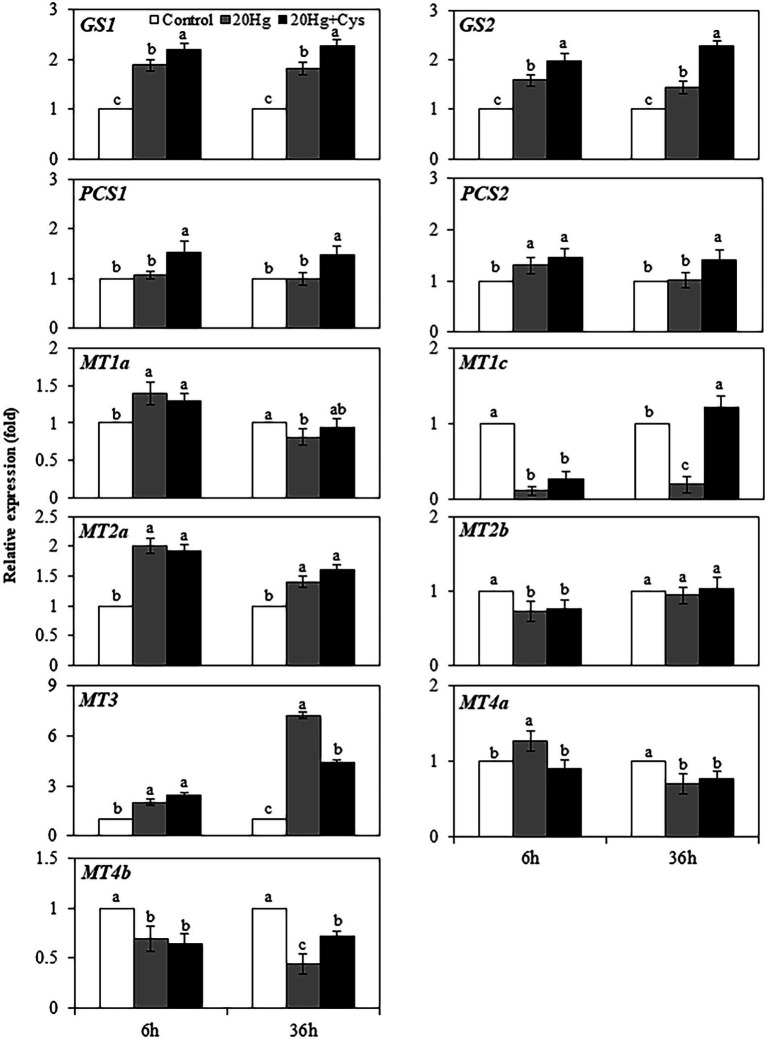
Effects of exogenous Cys on the expression levels of heavy metal chelators in *Arabidopsis* seedlings under Hg stress. Data represent the mean ± SD of three independent replicates. Different letters indicate significant differences (*p* < 0.05).

Heavy metal chelators, such as GSH, PC, and MT, play crucial roles in reducing the intracellular toxicity of free Hg ions through chelation. As Cys itself is a major component of GSH, PC, and MT, we determined the effects of exogenous Cys supplementation on the regulation of genes related to the synthesis of these three chelators in *Arabidopsis* seedlings under Hg stress. Transcript levels of all genes were downregulated by 6 and 36 h of Cys treatment (Supplementary Figure 3) but exhibited varied changes by Hg or Hg + Cys treatment (Figure 4). The *GS1* and *GS2* transcript levels, which were slightly increased after 6 and 36 h of Hg exposure, were further increased in the presence of exogenous Cys. However, no significant changes in *PCS1*, *PCS2*, *MT1a*, *MT2b*, and *MT4a* transcript levels were found in the Hg or Hg + Cys groups at both times. Although the level of *MT1c* expression was greatly reduced under the Hg and Hg + Cys treatment conditions at 6 h, the transcript level was recovered to the control level after 36 h in the presence of exogenous Cys. *MT2a* expression, which was induced under both Hg and Hg + Cys treatment conditions, was higher in level at 6 h than at 36 h. *MT3* expression, which was increased by approximately 2-fold under both Hg and Hg + Cys treatment conditions at 6 h, was further increased by approximately 7-fold and 4-fold, respectively, after 36 h. By contrast, the continuous reduction in *MT4b* expression was observed at both times under both Hg and Hg + Cys treatment conditions.

### Effects of Exogenous Cys on Transcript Levels of Heavy Metal Chelators in Roots and Shoots Under Hg Stress

**Figure 5:**
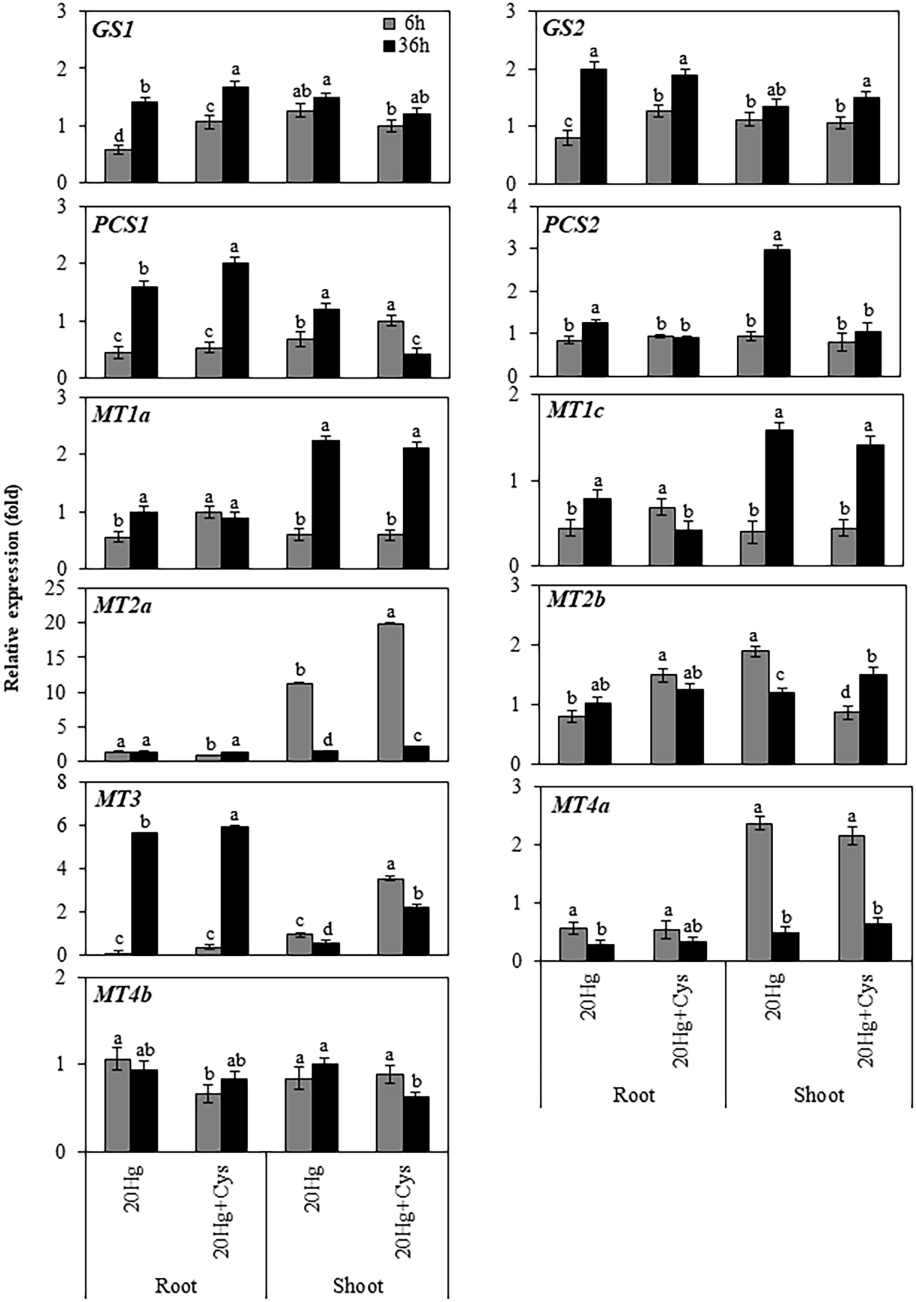
Effects of exogenous Cys on the expression levels of heavy metal chelators in roots and shoots of *Arabidopsis* under Hg stress. Data represent the mean ± SD of three independent replicates. Different letters indicate significant differences (*p* < 0.05).

Having found that a high concentration of Hg was accumulated in *Arabidopsis* roots, we next determined the transcript levels of heavy metal chelators in the roots and shoots of this plant (Figure 5). The transcript levels of *GS1* and *GS2* in the roots increased in a time-dependent manner under both Hg and Hg + Cys treatment conditions, whereas no significant alterations in the levels were found in the shoots. *PCS1* expression in the roots decreased rapidly within 6 h and then increased at 36 h under both Hg and Hg + Cys treatments, whereas that in the shoots showed time dependent increase and decrease under Hg and Hg + Cys, respectively. There was no significant change in the levels of *PCS2* transcript under either treatment condition in the roots, but they were significantly induced after 36 h of Hg stress in the shoots. Similar expression patterns were found for *MT1a* and *MT1c* in the roots and shoots, where under both treatment conditions, neither heavy metal chelator was induced in the roots, but both were upregulated in the shoots at 36 h. Notably, 6 h of Hg stress markedly induced the expression of *MT2a* (11-fold) in the shoots, and exogenous Cys further increased this up to 20-fold, whereas there were no changes observed in the roots under both treatment conditions (Figure 5). By contrast, *MT3* expression in the roots was greatly decreased within 6 h and then markedly induced (6-fold) after 36 h under both Hg and Hg + Cys treatment conditions. In the shoots, Hg caused a reduction in the *MT3* transcript level, whereas its expression was slightly increased (by upto 2-fold) in the presence of exogenous Cys at 6 h and was higher than that at 36 h. Under both Hg and Hg + Cys treatment conditions, the *MT4a* expression level was decreased in the roots but showed a 2-fold induction at 6 h and a reduction at 36 h in the shoots. By contrast, no significant induction of *MT2b* or *MT4b* expression was found in both the roots and shoots under any treatment condition.

### Effects of Exogenous Cys on H_2_O_2_ Production and Cell Damage Under Hg Stress

**Figure 6:**
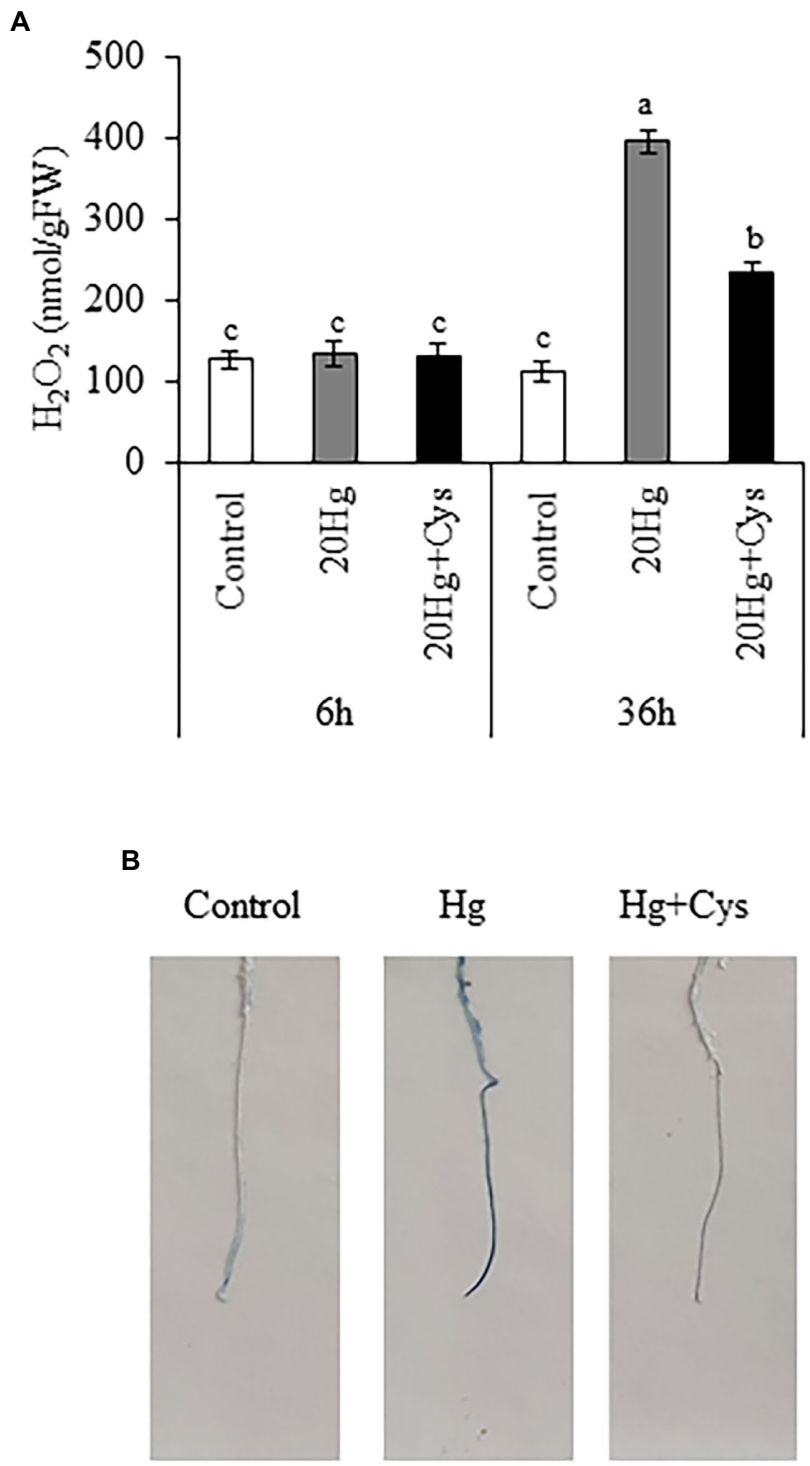
Effects of exogenous Cys on the H_2_O_2_ level in seedlings **(A)** and cell damage in roots **(B)** of *Arabidopsis* under Hg stress. **(A)** H_2_O_2_ levels were determined in *Arabidopsis* seedlings treated with 20 μM Hg with or without 50 μM Cys for 6 and 36 h. Data represent the mean ± SD of three independent replicates. Different letters indicate significant differences (*p* < 0.05). **(B)** Visualization of cell death in roots of *Arabidopsis* seedlings by Evans blue staining.

The production of H_2_O_2_ was examined in *Arabidopsis* seedlings, where it was found that 20 μM Hg did not lead to the production of H_2_O_2_ at 6 h, but it did markedly elevate the level after 36 h (1.98-fold; Figure 6A). Exogenous Cys significantly decreased the Hg-induced H_2_O_2_ content to a similar level as the control after 36 h. Next, we determined the effect of Cys on Hg-induced root cell damage using Evans blue staining, which indicates the degree of oxidation in plant cells (Figure 6B). An obvious increase in staining density was observed in the roots of the Hg-treated group. By contrast, the roots in the Hg + Cys-treated group displayed a much lower density of staining, being similar to the level of the control. These results indicate that exogenous Cys application can alleviate the Hg-induced accumulation of H_2_O_2_, thereby mitigating the oxidative damage in the root cells.

### Effects of Exogenous Cys on Transcript Levels of Antioxidative Enzymes in Seedlings Under Hg Stress

**Figure 7:**
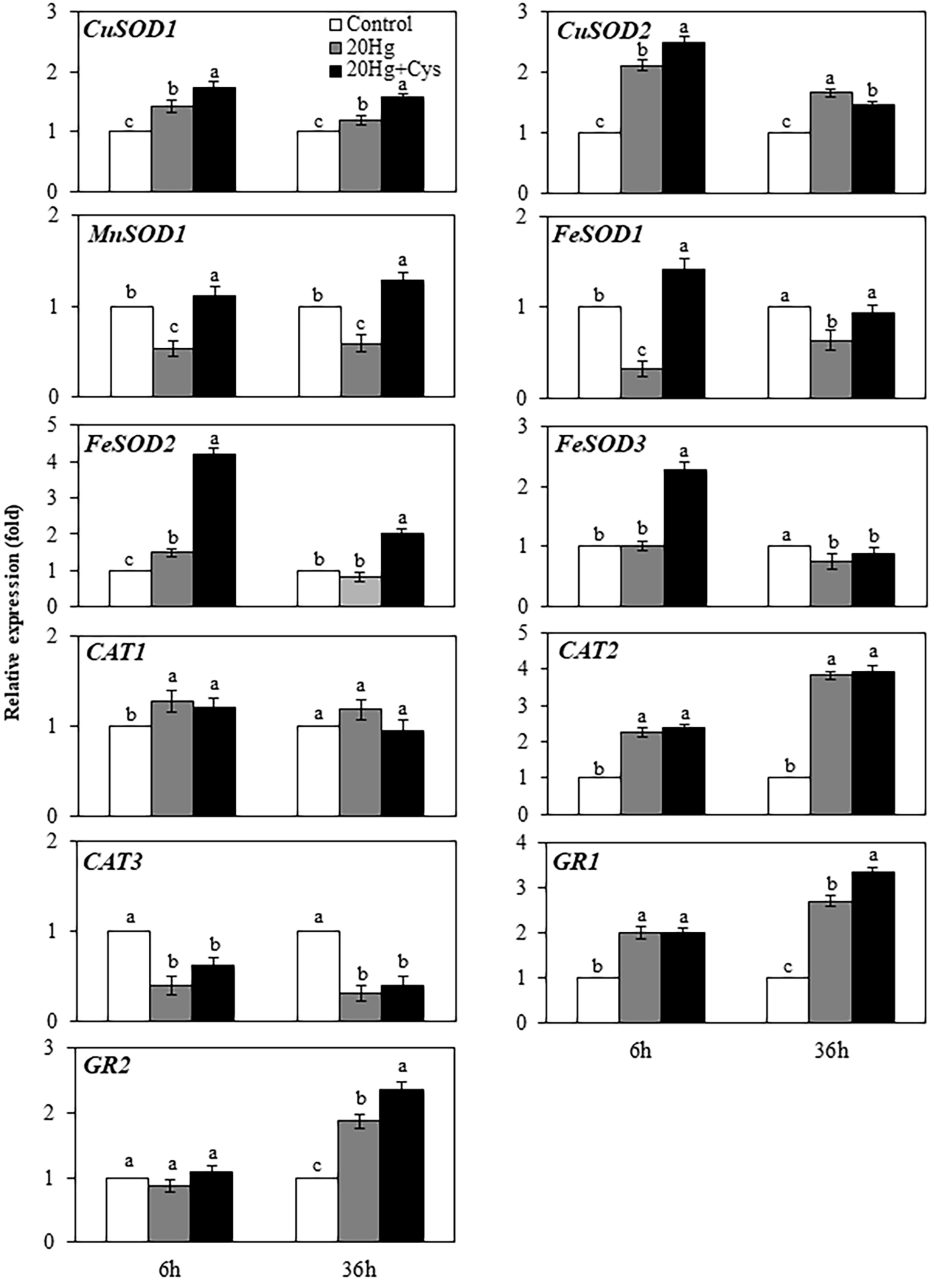
Effects of exogenous Cys on the expression levels of antioxidative enzymes in *Arabidopsis* seedlings under Hg stress. Data represent the mean ± SD of three independent replicates. Different letters indicate significant differences (*p* < 0.05).

Given the importance of antioxidative enzymes in scavenging ROS, we determined the transcript levels of the main enzymatic antioxidants (including SOD, CAT, and GR) in the Hg- and Hg + Cys-treated groups of *Arabidopsis* seedlings (Figure 7). At 6 h treatment, Hg had induced the expression of *CuSOD1* and *CuSOD2* slightly, and the levels were marginally increased in the presence of exogenous Cys, with the expression level being higher for *CuSOD2* than for *CuSOD1*. However, no significant changes in the expression levels of *CuSOD1* and *CuSOD2* were observed at 36 h of either Hg or Hg + Cys treatment. *MnSOD1* and *FeSOD1* exhibited similar expression patterns to each other, with the Hg-induced decreases in the levels of both enzymes being recovered to the control level by exogenous Cys treatment at both times. Hg treatment alone did not alter the transcript levels of *FeSOD2* and *FeSOD3* at both 6 and 36 h, whereas exogenous Cys induced the expression of these two enzymes by 4-fold and 2-fold, respectively, within 6 h and then significantly decreased the levels at 36 h. Neither the Hg nor the Hg + Cys treatments altered the transcript level of *CAT1* but they did decrease that of *CAT3* at both times. In the case of *CAT2* and *GR1*, both the Hg and Hg + Cys treatments increased the induction of the enzymes by 2-fold at 6 h and further increased them after 36 h, with exogenous Cys increasing the transcript level of *GR1* slightly more than Hg at this latter time point. No significant changes in *GR2* expression levels were observed after Hg or Hg + Cys treatments at 6 h, whereas the levels were increased at 36 h in both treatment groups. Like that of *GR1*, the mRNA expression of *GR2* was increased more by exogenous Cys than by Hg alone.

### Effects of Exogenous Cys on Transcript Levels of Antioxidative Enzymes Under Hg Stress in *Arabidopsis* Roots and Shoots

Next, we determined the transcript patterns of the antioxidative enzymes in the roots and shoots of *Arabidopsis* (Table 1). The transcript levels of all enzymatic antioxidant genes in the roots were quickly reduced by Hg-induced oxidative stress within 6 h and further increased by Cys application. In particular, *GR1* showed the highest expression level of all the enzymatic antioxidant genes in the presence of Cys. At 6 h, no significant transcript changes in *CuSOD1*, *CuSOD2*, *MnSOD1*, *CAT1*, and *GR1* were observed in the shoots in the presence of Hg or Hg + Cys. However, Hg marginally reduced the transcript levels of *FeSOD1*, *FeSOD2*, and *FeSOD3* and markedly reduced those of *CAT2* and *GR2*, but these levels were not altered by the exogenous Cys treatment. Among the enzymatic antioxidant genes, only *CAT3* showed very slight induction (about 1.8-fold) under both Hg and Hg + Cys treatments. These results indicate that treatment with Cys for a short time can upregulate the expression of all enzymatic antioxidant genes in the roots, but those in the shoots are not affected.

**Table 1:** Effect of exogenous 50 μM Cys on transcript levels of antioxidative enzymes in roots and shoots of *Arabidopsis* under Hg stress.

Treatment	6 h				36 h			
	Root		Shoot		Root		Shoot	
	Hg	Hg + Cys	Hg	Hg + Cys	Hg	Hg + Cys	Hg	Hg + Cys
CuSOD1	0.42 ± 0.09^c^	1.41 ± 0.11^a^	0.93 ± 0.09^b^	1.16 ± 0.11^b^	1.23 ± 0.14^b^	1.26 ± 0.16^b^	1.19 ± 0.08^b^	2.66 ± 0.15^a^
CuSOD2	0.71 ± 0.11^b^	1.12 ± 0.12^a^	0.91 ± 0.11^ab^	0.94 ± 0.12^ab^	1.16 ± 0.06^b^	1.26 ± 0.11^b^	0.94 ± 0.13^c^	1.68 ± 0.10^a^
MnSOD1	0.35 ± 0.11^c^	0.84 ± 0.16^b^	1.06 ± 0.11^a^	1.12 ± 0.16^a^	2.00 ± 0.09^a^	2.03 ± 0.10^a^	0.50 ± 0.11^b^	2.13 ± 0.10^a^
FeSOD1	0.13 ± 0.06^c^	0.63 ± 0.05^a^	0.60 ± 0.08^a^	0.50 ± 0.10^b^	1.39 ± 0.14^a^	1.40 ± 0.11^a^	1.28 ± 0.16^a^	0.92 ± 0.05^b^
FeSOD2	0.40 ± 0.09^c^	1.12 ± 0.10^a^	0.75 ± 0.13^b^	0.79 ± 0.10^b^	0.94 ± 0.09^a^	0.91 ± 0.10^a^	1.06 ± 0.09^a^	1.06 ± 0.10^a^
FeSOD3	0.84 ± 0.10^b^	1.19 ± 0.08^a^	0.75 ± 0.12^b^	0.71 ± 0.11^b^	0.66 ± 0.10^b^	0.75 ± 0.12^b^	1.19 ± 0.10^a^	0.94 ± 0.08^a^
CAT1	0.25 ± 0.11^c^	0.71 ± 0.10^b^	1.16 ± 0.13^a^	0.94 ± 0.10^ab^	0.77 ± 0.11^c^	0.83 ± 0.10^c^	1.33 ± 0.15^a^	1.12 ± 0.10^b^
CAT2	0.32 ± 0.08^b^	0.80 ± 0.05^a^	0.33 ± 0.10^b^	0.24 ± 0.13^b^	0.86 ± 0.13^a^	0.87 ± 0.12^a^	0.94 ± 0.08^a^	0.45 ± 0.05^b^
CAT3	0.45 ± 0.09^c^	0.75 ± 0.10^b^	1.88 ± 0.10^a^	1.78 ± 0.09^a^	0.92 ± 0.12^ab^	0.96 ± 0.10^ab^	1.12 ± 0.09^a^	0.53 ± 0.10^b^
GR1	0.86 ± 0.09^b^	2.00 ± 0.11^a^	0.94 ± 0.12^b^	0.87 ± 0.10^b^	2.00 ± 0.08^a^	2.24 ± 0.11^a^	1.68 ± 0.08^b^	1.78 ± 0.11^b^
GR2	0.38 ± 0.11^c^	0.84 ± 0.10^a^	0.50 ± 0.11^b^	0.53 ± 0.12^b^	0.94 ± 0.13^b^	1.06 ± 0.10^b^	1.26 ± 0.13^a^	0.89 ± 0.10^b^

CAT, catalase; Cys, cysteine; GR, glutathione reductase; Hg, mercury; SOD, superoxide dismutase. Data represent the mean ± SD of three independent replicates. Different letters indicate significant differences (*p* < 0.05).

In contrast to the effects at 6 h, 36 h of Hg exposure did not affect the transcript levels of *CuSOD1*, *CuSOD2*, *FeSOD1*, *FeSOD2*, *CAT2*, *CAT3*, and *GR2* in the roots, but it did increase those of *MnSOD1* and *GR1* and decreased those of *FeSOD3* and *CAT1*. At this time, exogenous Cys treatment did not affect the transcript levels of all examined genes in the roots. Under Hg or Hg + Cys treatment conditions, gene regulation was found to vary in the shoots, in that Hg did not alter the transcript levels of *CuSOD1* and *CuSOD2* but reduced that of *MnSOD1*, whereas the expression levels of all three genes were significantly increased by exogenous Cys treatment. By contrast, no significant changes in *FeSOD1*, *FeSOD2*, *FeSOD3*, and *CAT1* transcript levels were observed under both treatment conditions. Although Hg did not affect *CAT2, CAT3,* and *GR2*, their expression was downregulated by exogenous Cys. *GR1* expression in the shoots was slightly induced by the Hg and Hg + Cys treatments, similar to the results in the roots.

## Discussion

Plants have evolved multiple adaptation mechanisms to protect themselves under Hg stress. Despite the accumulating evidence emphasizing the crucial roles of Cys-containing heavy metal chelators in plant responses to Hg stress, the effect of exogenous application of Cys on Hg tolerance has not been reported in *Arabidopsis*. We demonstrated that exogenous Cys markedly alleviated the inhibition of germination and seedling growth caused by Hg stress in *Arabidopsis*. Unlike the results of Aysin et al. (2020), which indicated that Cys treatment decreased Hg accumulation and tolerance in maize seedlings, our findings showed that exogenous Cys markedly enhanced Hg accumulation in both, seedlings and *Arabidopsis* plants at the reproductive phase under short and long periods of Hg exposure (Figure 2A). These differences in results suggest that exogenous Cys regulates Hg uptake differently depending on the plant species. Our NMR data clearly proved that Cys has strong Hg-binding affinity, with the –SH group of Cys quickly and specifically binding to Hg, as indicated by the large downshifts of the H1 and H2 protons located in this functional group within a few minutes (Supplementary Figure 2). Even though GSH has also been shown to have strong Hg-specific binding affinity, exogenous GSH decreases Hg accumulation by inhibiting its uptake in *Arabidopsis* (Kim et al., 2017), indicating that Cys and GSH possess different Hg uptake mechanisms. It is obvious that Cys, like GSH, could bind rapidly to Hg outside the roots (Kim et al., 2017), and the Hg–Cys complex then enters by crossing the root cell membrane, resulting in the high Hg accumulation in *Arabidopsis*. Interestingly, the entered Hg–Cys complex hardly translocate towards the shoot but retained in the root cells (Figure 2B), suggesting that the Hg accumulation in the roots occupies most amount of Hg in the whole plants. Unlike when Cys exists, in the Hg alone treated Arabidopsis, it is possible that Hg tightly binds to thiol residues of proteins in root epidermal cell walls (Sobrino-Plata et al., 2021), resulting in the increasing Hg accumulation in the roots.

The next important question is how Cys expedites Hg absorption into the *Arabidopsis* roots. Given that the Hg–Cys complex could be transported by Cys transporters present in the root cells, the modulation of such transporters during Hg exposure should be investigated. However, there is no information in the current literature about Cys transporters in plants. Because previous studies has suggested that *AtAAP1* and *AtLHT1* might play roles in the uptake of neutral amino acids by *Arabidopsis* roots (Himer et al., 2006; Lee et al., 2007; Perchlik et al., 2014), we investigated whether these amino acid transporters are also involved in Hg uptake into *Arabidopsis* roots, given that Cys belongs to the family of neutral amino acids. Interestingly, Cys supplementation markedly increased the expression level of *AtLHT1* but not that of *AtAAP1* in the roots (Figure 3). It is likely that *AtLHT1* actively participates in the uptake of the Hg–Cys complex and facilitates higher Hg accumulation in the roots. To clarify more clearly role or mechanism of exogenous Cys on the absorption and sequestration of Hg–Cys complex into the roots, it is needed to be further studied cellular Cys analysis using the mutants or transgenic plants of Cys transporters.

Because of their strong affinity for the –SH functional groups in essential biomolecules, intracellular free Hg ions can alter the conformation of proteins and affect various metabolic pathways (Ajsuvakova et al., 2020). Numerous studies have proved important roles of Hg chelation by various heavy metal chelators to reduce the deleterious effect of Hg in cells (Ajsuvakova et al., 2020; Natasha et al., 2020). Even though PC is known to one of major chelator, Hg did not induce *PCS* in seedlings (Figure 4). PCS are usually constitutively expressed and biosynthesis of PCs is activated at post-translational level by heavy metals, and thus PC may partially play role in Hg detoxification despiting of no induction of PCS. Hg also upregulated *GS1*, *GS2*, *MT2a*, and *MT3*, implying that Hg stimulates the transcriptional regulation for synthesis of GSH and MTs. Recent study has shown that exogenous Cys increased the GSH content of maize seedlings in presence of Hg (Aysin et al., 2020). Our results also revealed that exogenous Cys further increased the expression of GSH synthesis related genes *GS1* and *GS2*. Therefore, it is obvious that exogenous Cys stimulates GSH synthesis only rather than MT and PC synthesis as the predominant pathway for Hg chelation in the seedlings.

It is widely reported that a higher amount of Hg is retained in the roots than in the shoots of many plants (Cho and Park, 2000; Ericksen and Gustin, 2004; Wang et al., 2015; Ahammad et al., 2018; Sobrino-Plata et al., 2021). Having found in this study that Hg was also accumulated mainly in the *Arabidopsis* roots in the presence of exogenous Cys (Figure 2B), we were interested to investigate the difference between the roots and shoots in terms of their regulation of the heavy metal chelator transcripts. Noticeable induction of *MT3* in the roots was observed with slight upregulation of *GS2* and *PCS1* at late period of Hg exposure (Figure 5), suggesting that MT3 is the crucial gene that confers diminished Hg toxicity, with the cooperation of *GS2* and *PCS1* occurring at the late stage of Hg stress. It has recently been reported that 3 μM Hg increased PC synthesis but decreased GSH accumulation in *Arabidopsis* roots (Sobrino-Plata et al., 2021). These results suggest that PC synthesis pathway is important response against Hg stress in *Arabidopsis* roots but GSH synthesis is depending on dose of Hg exposure. Previous study reported that exogenous Cys declined PC synthesis but increased GSH contents in roots of *Iris lactea* var. *chinensis* in the presence of Pb (Yuan et al., 2015). Unexpectedly, exogenous Cys did not induce the expression of any of these genes significantly in the roots at all Hg exposure periods. It can be speculated that a high amount of Hg may exist in the form of Hg–Cys complexes in the roots, as revealed by the NMR analysis, resulting in the alleviation of Hg toxicity without induction of other heavy metal chelators. In contrast to the roots, the shoots exhibited more rapid and varied changes in the expression of the *MT* genes. Notably, exogenous Cys remarkably upregulated Hg-induced *MT2a* expression after 6 h of Hg exposure in the shoots. Furthermore, it also increased *MT3* expression, albeit at a much lower level than that of *MT2a*. These results indicate that exogenous Cys can promote a higher level of MT synthesis than it does GSH and PC synthesis during a short period of Hg exposure and could confer Hg detoxification mainly through MT2a with the assistance of MT3 in the shoots.

Once Hg is absorbed by the root cells, the Hg ions trigger ROS production and membrane lipid peroxidation, resulting in metabolic disorders and serious cellular injuries in plants (Israr et al., 2006; Ortega-Villasante et al., 2007; Kim et al., 2017; Natasha et al., 2020). In this study, after long Hg exposure time, H_2_O_2_ production significantly increased in *Arabidopsis* seedlings, leading to cell damage, which is consistent with the findings of another study (Kim et al., 2017). However, exogenous Cys supplementation effectively suppressed the H_2_O_2_ amount and cell damage (Figure 6). Similar positive effects of exogenous Cys were also observed in the maize seedlings under Hg stress (Aysin et al., 2020). Therefore, Cys maybe directly involved in ROS scavenging, thereby promoting seedling growth under Hg-induced oxidative stress. ROS scavenging is accompanied by an antioxidative defense system that comprises both enzymatic and nonenzymatic antioxidants, which maintain the redox state in plants. It has previously been shown that exogenous Cys increases the expression of *SOD* and *CAT*, but not *GR*, under Hg stress in maize seedlings (Aysin et al., 2020). Our results also revealed a variable modulation of the transcript levels of various enzymatic antioxidants by exogenous Cys depending on the exposure period. In seedlings, exogenous Cys rapidly increased the expression of all *SOD* genes (*viz.*, *CuSOD1*, *CuSOD2*, *MnSOD1*, *FeSOD1*, *FeSOD2*, and *FeSOD3*) within 6 h relative to the levels produced in the presence of Hg alone (Figure 7). Kim et al. (2017) had demonstrated that Hg significantly increased the level of O_2_^−^ in *Arabidopsis* seedlings. Taken together, these results suggest that the Hg-induced promotion of O_2_^−^ production in *Arabidopsis* seedlings could be efficiently quenched by the exogenous Cys-induced SODs, thereby mitigating excess O_2_^−^ toxicity. However, 36 h of exogenous Cys exposure further increased the Hg-induced upregulation of *GR1* and *GR2*, which could efficiently eliminate the excess production of H_2_O_2_ promoted by Hg-induced oxidative stress at the late stage. These results matched the higher expression levels of H_2_O_2_-scavenging enzymes and the lower H_2_O_2_ content in the presence of Cys.

Previous studies have reported that the activities and expression levels of enzymatic antioxidants were regulated differently between roots and shoots in response to Hg stress (Zhou et al., 2007, 2008; Sobrino-Plata et al., 2009; Manikandan et al., 2015; Wang et al., 2015; Cruz et al., 2021). In this study, different patterns of modulation of the antioxidative enzymes were also found between the roots and shoots in the presence of Hg or Hg + Cys (Table 1). Short-time Hg exposure resulted in more significantly reduced levels of antioxidative enzyme expression in the roots than in the shoots, implying that Hg-induced oxidative stress directly affects the root cells and thus weakens the enzymatic antioxidative defense system in the roots, thereby disrupting cellular metabolism more severely in this plant organ than in the shoots. The Cys upregulation of the antioxidative enzyme expression levels that had been reduced by Hg stress provides clear evidence that the exogenous application of Cys can activate these enzymatic antioxidants, thereby conferring to the plant the ability to mitigate the oxidative cell damage caused by intracellular Hg in the roots. In contrast to the short-time exposure of exogenous Cys, its long-time exposure significantly induced the expression of *CuSOD1*, *CuSOD2*, and *MnSOD1* in the shoots but did not alter any of the enzymatic antioxidant genes in the roots, suggesting that its role in the shoots is more to scavenge O_2_^−^ as a late response. Taken together, the results indicate that exogenous Cys functions to scavenge ROS rapidly in the roots, but does this later in the shoots, in response to Hg-induced oxidative stress. However, further studies are needed to fully elucidate the role of Cys in the intracellular fate of Hg, such as Hg uptake and transportation through other candidate transporters and the distribution of Hg in the different plant cell organelles.

In conclusion, we have shown that exogenous Cys confers the capacity for Hg tolerance in *Arabidopsis* even though it also facilitates high Hg accumulation in the plant. Exogenous Cys could promote Hg uptake *via* the Cys transporter AtLHT1 and sequester Hg ions in the roots. Furthermore, exogenous Cys modulated the transcript levels of LMW thiols and antioxidative enzymes, with the regulatory patterns varying among the seedlings, roots, and shoots of *Arabidopsis*, indicating that Cys likely reduces intracellular Hg toxicity by promoting chelation of Hg ions and ROS scavenging. Our study reveales the positive functional roles and the molecular mechanism of exogenous Cys on the Hg uptake and tolerance in *Arabidopsis* and provides a new strategy for the phytoremediation of Hg-contaminated soils.

## Data Availability Statement

The datasets presented in this study can be found in online repositories. The names of the repository/repositories and accession number(s) can be found in the article/Supplementary Material.

## Author Contributions

Y-OK designed the experiments and wrote the manuscript. JK and Y-OK supervised the research and revised the manuscript. Y-OK and YG performed the experiments and data analysis. All authors contributed to the article and approved the submitted version.

## Funding

This research was supported by a grant from the Basic Science Research Program through the National Research Foundation (NRF) of Korea, funded by the Ministry of Education (NRF-2018R1D1A1B07045677).

## Conflict of Interest

The authors declare that the research was conducted in the absence of any commercial or financial relationships that could be construed as a potential conflict of interest.

## Publisher’s Note

All claims expressed in this article are solely those of the authors and do not necessarily represent those of their affiliated organizations, or those of the publisher, the editors and the reviewers. Any product that may be evaluated in this article, or claim that may be made by its manufacturer, is not guaranteed or endorsed by the publisher.
