# Supplementary material for: Exogenous Cysteine Improves Mercury Uptake and Tolerance in Arabidopsis by Regulating the Expression of Heavy Metal Chelators and Antioxidative Enzymes
Source: Front Plant Sci. 2022 Jun 10;13:898247. doi: 10.3389/fpls.2022.898247 (PMC9231614; doi:10.3389/fpls.2022.898247)
Supplement: Supplementary file 1 [file Table_1.DOCX]

Supplementary Material

Exogenous Cysteine Improves Mercury Uptake and Tolerance in *Arabidopsis* by Regulating the Expression of Heavy Metal Chelators and Antioxidative Enzymes

Supplementary Figures

**
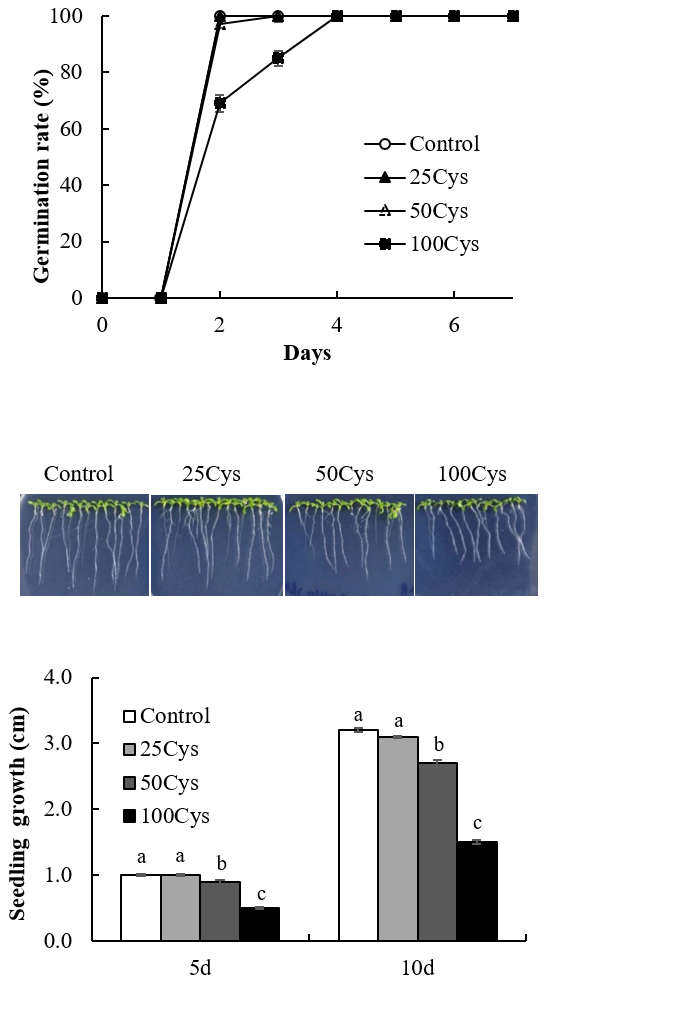
**

**Supplementary Figure 1.** Effects of Cys on *Arabidopsis* seed germination and seedling growth*.*


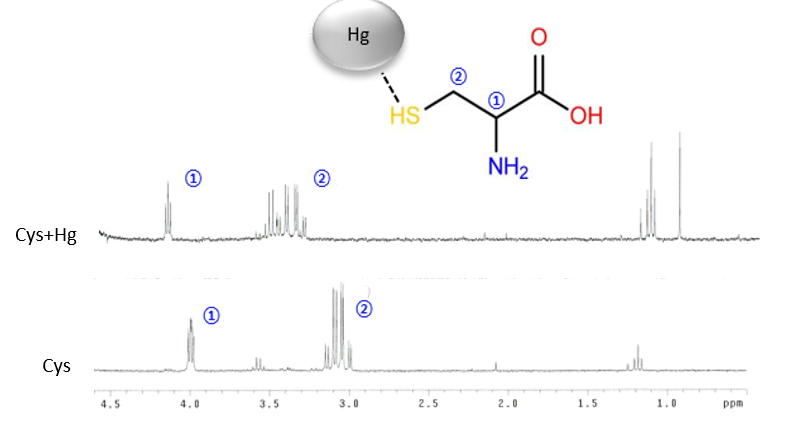


Supplementary Figure 2. ^1^H NMR spectra of Cys with 1 equivalent of Hg^2+^ (numbers indicating peaks correspond to protons in the chemical formula indicated with those numbers).


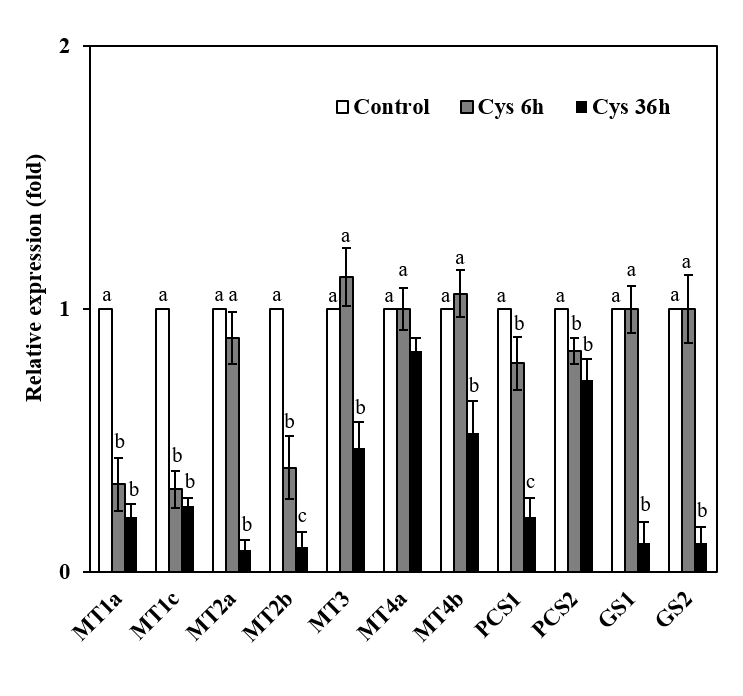


Supplementary Figure 3. Effects of exogenous Cys on the expression levels of heavy metal chelators in *Arabidopsis* seedlings.

**Supplementary Table 1.** List of primers used in the qRT-PCR analysis of heavy metal chelator genes of *Arabidopsis*.

| **Gene** | **Forward primer (5ʹ to 3ʹ)** | **Reverse primer (5ʹ to 3ʹ)** |
| --- | --- | --- |
| *AtPCS1*  *AtPCS2*  *AtGS1*  *AtGS2* | TGGTGACTGGAGTTGTGGTG  TTGACCCGGGAAGAAAGTGG  AGCATGTCGCTGAAGATGTC  GTTCGTTTCCTGGCCTTAGTC | CCCTGACCATGTCTGTGGAG  TTTGTGCGGAAAGCTTCGAC  AACACGGGATCTACGCTTTG  CCATGACTACCGCTCTTGGG |
| *AtMT1a* | CCTGCAAATGTGGTGACTCT | ACCCACAGCTGCAGTTTGAT |
| *AtMT1c* | CCTGCAAATGTGGTGATTCGT | ACAGTTACAGCTTGACCCGCA |
| *AtMT2a* | GCGAGACAACCACAACTGAG | TGCAGGTGCAAGGATCACAC |
| *AtMT2b* | GTGGAAGCTGTGGTTGTGG | AACGAAAGTCTCGCCGGAAG |
| *AtMT3* | TGCTGACAAGACCCAGTGC | GCAAGTGCAGTTGACGCAG |
| *AtMT4a* | ATCTGAATGTGGCAGAGGGAC | CATGTGCCCTTGATCCCCAG |
| *AtMT4b* | TGCAGGTTAATGATGAGCG | CAAGTGCAACCCTTAGCAG |

**Supplementary Table 2.** List of primers used in the qRT-PCR analysis of antioxidative enzyme genes of *Arabidopsis*.

| **Gene** | **Forward primer (5ʹ to 3ʹ)** | | **Reverse primer (5ʹ to 3ʹ)** | |
| --- | --- | --- | --- | --- |
| *Cu/ZnSOD1* | GGTCCACATTTCAACCCCGA | CTGCATGGACAACAACAGCC | |  |
| *Cu/ZnSOD2*  *MnSOD1*  *FeSOD1*  *FeSOD2*  *FeSOD3*  *CAT1*  *CAT2*  *CAT3*  *GR1*  *GR2*  Actin | GATGGCGTGGCAGAAACAAC  AATTCAACGGCGGAGGTCAT  TCACCGCAAACTACGTCCTC ACGGTTAGGGACAAAGGTGG  GGATGTGTGGGAGCACTCTT  ACTACAGGCACATGGAAGGC  GCTCGATGTGACCAAGACCT  ACATGGAGGGTTTCGGTGTC  TCTGGACGCCAGGAAAAGAC  TGGGCTGTTGGGGATGTTAC  CAGCAGAGCGGGAAATTGTAAGAG | CCACCCTTTCCGAGGTCATC  GCACCCTCAGCACTCATCTT  GAGCAGCGTTGTTGAAAGCA AATCCAAGGTTTCCCGGCTC  AGCGATTGGGATGTTGGGTT  ATGGCTGTGATTGGCTCCTC  GGCATAGGAGAAGACACGGG  TTAGTGGCGTGGCTGTGATT  TGGATGTATCCCAACCGTGC  TCCAATAGGTGGCTGGGAGA  TTCCTTTCAGGTGGTGCAACGAC | |  |

**Supplementary Table 3.** Effects of exogenous Cys on *Arabidopsis* postgerminative seedling growth and root elongation under Hg stress.

|  | **Postgerminative seedling growth (cm)** | | |  | **Root length after treatments (cm)** | | |
| --- | --- | --- | --- | --- | --- | --- | --- |
| **Treatments** | **5d** | **7d** | **10d** |  | **5d** | **7d** | **10d** |
| Control | 2.32± 0.21 | 3.81 ± 0.1 | 5.55 ± 0.1 |  | 1.76 ± 0.01 | 3.15 ± 0.02 | 5.17 ± 0.02 |
| 10Hg | 0.81 ± 0.13 | 1.26 ± 0.1 | 1.52 ± 0.2 |  | 0.23 ± 0.00 | 1.38 ± 0.01 | 2.11 ± 0.02 |
| 10Hg+50Cys | 1.92 ± 0.10 | 3.16 ± 0.1 | 3.92 ± 0.1 |  | 1.38 ± 0.05 | 2.75 ± 0.05 | 3.85 ± 0.05 |
| 20Hg | 0.51 ± 0.13 | 0.71 ± 0.05 | 1.08 ± 0.05 |  | 0.26 ± 0.05 | 0.76 ± 0.05 | 1.51 ± 0.05 |
| 20Hg+50Cys | 1.28 ± 0.13 | 1.83 ± 0.05 | 2.37 ± 0.05 |  | 0.96 ± 0.05 | 2.34 ± 0.05 | 3.09 ± 0.05 |
| 30Hg | 0.11 ± 0.10 | 0.32 ± 0.00 | 0.54 ± 0.00 |  | 0.15 ± 0.00 | 0.39 ± 0.00 | 0.53 ± 0.00 |
| 30Hg+50Cys | 1.14 ± 0.11 | 1.35 ± 0.05 | 1.78 ± 0.05 |  | 0.69 ± 0.05 | 1.15 ± 0.05 | 1.88 ± 0.05 |

Average values of 10 plants were considered as one replication and three independent biological replications were performed.
